# Cost-Effectiveness Analysis of 1-Year Treatment with Golimumab/Standard Care and Standard Care Alone for Ulcerative Colitis in Poland

**DOI:** 10.1371/journal.pone.0160444

**Published:** 2016-08-05

**Authors:** Ewa Stawowczyk, Paweł Kawalec, Andrzej Pilc

**Affiliations:** 1 StatSoft Polska Sp. z o.o., Krakow, Poland; 2 Drug Management Department, Institute of Public Health, Faculty of Health Sciences, Jagiellonian University, Krakow, Poland; 3 Department of Neurobiology, Institute of Pharmacology, Polish Academy of Sciences, Krakow, Poland; Laikon Hospital, GREECE

## Abstract

**Objective:**

The objective of this study was to assess the cost-effectiveness of induction and maintenance treatment up to 1 year of ulcerative colitis with golimumab/standard care and standard care alone in Poland.

**Methods:**

A Markov model was used to estimate the expected costs and effects of golimumab/standard care and a standard care alone. For each treatment option the costs and quality adjusted life years were calculated to estimate the incremental cost-utility ratio. The analysis was performed from the perspective of the Polish public payer and society over a 30-years time horizon. The clinical parameters were derived mainly from the PURSUIT-SC and PURSUIT-M clinical trials. Different direct and indirect costs and utility values were assigned to the various model health states.

**Results:**

The treatment of ulcerative colitis patients with golimumab/standard care instead of a standard care alone resulted in 0.122 additional years of life with full health. The treatment with golimumab/standard care was found to be more expensive than treatment with the standard care alone from the public payer perspective and from social perspective. The incremental cost-utility ratio of golimumab/standard care compared to the standard care alone is estimated to be 391,252 PLN/QALY gained (93,155 €/QALYG) from public payer perspective and 374,377 PLN/QALY gained (89,137 €/QALYG) from social perspective.

**Conclusions:**

The biologic treatment of ulcerative colitis patients with golimumab/standard care is more effective but also more costly compared with standard care alone.

## Introduction

Ulcerative colitis (UC) is an idiopathic inflammatory bowel disorder characterized by an inflammatory reaction involving the colonic mucosa [1,2]. The clinical course is unpredictable and marked by alternating periods of exacerbation and remission, which may occur spontaneously or in response to environmental, psychosocial or treatment changes or intercurrent illnesses, as well as other medical factors influencing disease status [3,4]. Although progress has been made in the overall management of the disease, no medical cure has been discovered [5]. The therapy of ulcerative colitis is directed at quickly resolving symptoms and subsequently maintaining symptom-free periods. Lifelong medical treatment is required, and sometimes, when there is no alternative treatment option and the disease is very severe, the surgery is performed. Conventional therapy comprises corticosteroids, aminosalicylates and drugs that affect the immune response. Ulcerative colitis has a significant impact on quality of life and daily activity, as patients experience loss of energy, negative self-image, social fear [4].

Until recently, surgery was the only remaining choice for severe chronic ulcerative colitis patients who failed standard treatment (i.e. cyclosporine, corticosteroids, 6-mercaptopurine, azathioprine), or when it was not tolerated. In rare cases, non-traditional therapies such as tacrolimus, and thalidomide have been used with varying degrees of success. The introduction of anti-tumor necrosis factor-alfa (anti-TNFα) treatment allowed a new option for the management of ulcerative colitis and is expected to decrease the rate of colectomies or at least to extend the time to surgery, compared with standard treatment. TNFα is a proinflammatory cytokine found at increased concentrations in the blood, colonic tissue and stools of ulcerative colitis patients [2]. Golimumab is a human monoclonal antibody which prevents the binding of TNFα to its receptors [6]. On the one hand clinical trials suggest that golimumab/standard care has superior efficacy compared to standard care alone in moderate to severe non-acute UC [7,8]. On the other hand, the use of biologics constitutes a heavy burden for the public payer, so its usage can be limited in many countries.

In Poland, patients with severe UC who are not able to have ciclosporine therapy and don't respond to standard care have the possibility to receive the induction treatment with infliximab, which consists of 3 administrations of the drug. At present, there is no biological maintenance treatment of ulcerative colitis reimbursed in Poland, hence patients often lose their response or remission, which were achieved during the induction phase. Additionally, the lack of biological maintenance treatment leads to an increased rate of colectomies. In this connection, there was a need for economic evaluation of UC induction and maintenance therapy with a TNFα inhibitor at Polish settings.

This study uses an economic evaluation to assess the cost-effectiveness of induction and maintenance treatment up to 1 year of ulcerative colitis with golimumab/standard care and standard care alone in Poland.

## Materials and Methods

### Overview

**Fig 1:**
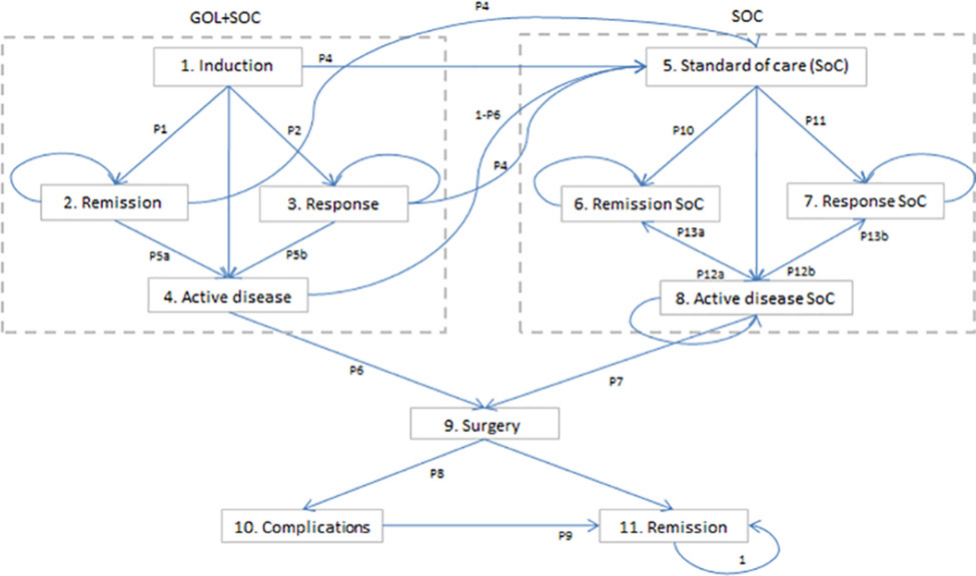
Structure of Markov model for patients with UC.

A Markov model was used to estimate the expected costs and effects of golimumab/standard care and standard care alone used in the induction and maintenance treatment of moderate to severe ulcerative colitis (model structure, inputs, transition probabilities, costs of health states, and utilities are presented in Fig 1, Tables 1 and 2). The analysis was taken from the perspective of the Polish public payer (National Health Fund, NHF) and also from social perspective (indirect costs included). Ulcerative colitis could be a lifelong disease, which is why the thirty-years’ time horizon was selected for the base-case analysis. Costs and outcomes were discounted at an annual rate of 5% and 3.5%, respectively.

**Table 1:** Clinical inputs and utilities used in the model.

Parameter		Value	95% LCI	95% UCI	Reference
Response rate—6. week	Golimumab/standard care (RR)	1.39	1.05	1.84	[7]
	Standard care alone	0.24	0.19	0.29	[7]
Remission rate—6. week	Golimumab/standard care (RR)	2.79	1.62	4.80	[7]
	Standard care alone	0.06	0.04	0.10	[7]
The probability of response per cycle—54. week	Standard care alone	0.014	0.008	0.023	[8]
Response per cycle—54. week (RR)	Golimumab/standard care	1.46	0.76	2.78	[8]
The probability of remission per cycle—54. week	Standard care alone	0.036	0.026	0.050	[8]
Remission per cycle—54. week (RR)	Golimumab/standard care	1.53	1.06	2.22	[8]
The probability of response loss per cycle—6–54. week	Golimumab/standard care	0.142	-	-	[8]
	Standard care alone	0.161	-	-	[8]
The probability of remission loss per cycle—6–54. week	Golimumab/standard care	0.000	-	-	[8]
	Standard care alone	0.000	-	-	[8]
The probability of complications after surgery		0.53	0.27	0.53	[10,11]
Surgery rate per cycle	Golimumab/standard care (RR)	0.77	0.33	1.86	[9]
	Standard care alone	0.75%	-	-	[9]
Utilities	Active treatment	0.420	0.320	0.420	[12,13]
	Remission	0.880	0.790	0.910	[12,13]
	Response	0.760	0.580	0.940	[12]
	Remission after surgery	0.610	0.610	0.610	[12]
	Complications after surgery	0.420	0.420	0.490	[12,13]

RR—relative risk; LCI—lower confidence interval; UCI—upper confidence interval

**Table 2:** Cost inputs used in the model.

Parameter		Mean	95% LCI	95% UCI	Reference
Drug cost [PLN]	Golimumab 1 mg	77.63	-	-	decree of the Minister of Health
	Azathioprine 1 mg	0.0107	-	-	
	Prednisolone 1 mg	0.1055	-	-	
	Mesalazine 1 mg	0.0015	-	-	
	Mercaptopurine 1 mg	0.0166	-	-	
Monitoring costs per cycle [PLN]	8 weeks cycle	121.56	-	-	Expert opinion, decree of the President of NHF
	1 week cycle	15.20	-	-	
Administration costs [PLN]		468.00	-	-	Expert opinion, decree of the President of NHF
Surgery cost [PLN]		12,480	6,240	37,440	Expert opinion, decree of the President of NHF
Complication after surgery treatment [PLN]		4,160	-	-	Expert opinion, decree of the President of NHF
Standard treatment per cycle [PLN]	8 weeks cycle	204.32	-	-	decree of the Minister of Health; expert opinion
	1 week cycle	25.54	-	-	
Indirect costs [PLN/year]	Remission	6,523.75	-	-	S1 File
	Active disease	22,934.58	-	-	

PLN—Polish zloty, €1 = 4.2 PLN, based on the average exchange course from the year 2015; NHF—National Health Fund

The target population consists of a hypothetical cohort of patients with an established diagnosis of UC and moderate to severe disease activity, defined as a Mayo score of 6–12, with an endoscopic subscore > 2 [7,8]. The cyclosporine therapy should be contraindicated or not tolerated by these patients. Additionally, according to Polish clinical practice, the patients have to meet at least one of the following criteria: (1) had an inadequate response to conventional therapies, including corticosteroids and 6-mercaptopurine or azathioprine (the total Mayo score > 6 points), (2) had failed to tolerate the therapy with corticosteroids and 6-mercaptopurine or azathioprine, (3) had contraindications to the therapy with corticosteroids and 6-mercaptopurine or azathioprine. The average age of a patient was 40.12 years (95% CI: 38.54–41.46), and the percentage of women was 46.0% (95% CI: 40.3–51.0%). This was based upon baseline data from the reference clinical trials the Program of Ulcerative Colitis Research Studies Utilizing an Investigational Treatment (PURSUIT)—Subcutaneous (SC) [7] and Maintenance (M) [8].

Based on the current practice, registered dosage and reference clinical trials, golimumab was assumed to be administered in a dose of 200 mg at week 0, 100 mg at week 2, and 50 mg every four weeks beginning at week 6 up to 1 year [6–8].

### Model structure

The modelling was carried out based on a Markov-type cohort simulation process and implemented in Microsoft Excel 2007 with Visual Basic for Applications tool (Microsoft Corporation, Redmond, WA). The patient enters this model when starting the induction treatment. The time horizon is divided into 2 periods: from week 0 to 6 (period 1; induction treatment), and from week 7 to the end of time horizon (period 2; maintenance treatment). The cycle length during period 1 is 1 week, and cycles in period 2 last 8 weeks. After the seventh cycle, the response to induction therapy and remission was assessed and biological treatment was continued only in responders (patients who responded or experienced remission). Mayo score was used to assess the UC activity (scores can range from 0 to 12, with higher scores indicating more severe disease activity): values from 0 to 2 means remission, 3–5 mild disease, 6–12 moderate-severe disease [7]. The clinical response was defined as a decrease from the baseline in the total Mayo score by at least 3 points and at least 30 percent, accompanied by either a rectal bleeding subscore of 0 or 1 or a decrease from baseline in rectal bleeding by at least 1. Clinical remission was defined as a Mayo score of 2 points or lower, with no individual subscore exceeding 1 point [7,8]. In accordance with Polish clinical practice, for standard care, it is assumed that in both the induction and maintenance phases, 100% of patients have corticosteroids and aminosalicylates, 80% have mercaptopurine, and 20% have azathioprine.

Patients who have neither remission nor response to induction treatment with the TNFα-inhibitor/standard care or standard care alone will stop the treatment, move to an active disease state and start standard care alone or will have a colectomy. If one of the treatments (golimumab/standard care or standard care alone) led to a clinical response or remission, the patient continued with the treatment in the maintenance phase.

Maintenance treatment with golimumab is restricted to 1 year in base-case analysis and no limitation for biological treatment was assumed in sensitivity analysis (golimumab administered until loss of response or death, according to what occurs first). Patients who experienced the response or remission can sustain or lose it during the next cycle. The treatment can be discontinued when unacceptable adverse events occur; in this case the patient moves to a standard care alone state. Patients who failed golimumab/standard care or standard care alone treatment continued standard care in the maintenance phase, but they could have a colectomy if their disease remained active. Standard care is continued regardless of whether the patient has remission, response or is in an active disease state. All patients who had a colectomy can experience temporary complications and finally achieve clinical remission after surgery (see: Fig 1). It was assumed that all complications occur immediately after surgery (in the same cycle) and are resolved during the 8-week period.

Certain adverse events were not included in the model. In accordance with reference clinical trials [7,8] the incidences of adverse events and serious adverse events, including serious infections, were similar for golimumab- and placebo-treated patients. No data was found on the significant impact of particular adverse events on the quality of life or costs.

There is no evidence, that patients with UC have lower life expectancy, thus the probability of death was calculated on a basis of life expectancy table for general Polish population (www.stat.gov.pl). It was assumed that the probability of death will be the same for each clinical states. No data was found on the different probability of death from particular clinical states of model for natural course of the disease.

### Clinical inputs

Transition probabilities in the model were calculated based on the response, remission rates and discontinuation due to adverse event rates which came from randomized, double-blind, placebo-controlled trials. The PURSUIT-SC and PURSUIT-M studies evaluated the efficacy of golimumab induction and maintenance therapy of patients with moderate to severe ulcerative colitis despite conventional treatment [7,8]. As the definitions of remission and response were overlapping in PURSUIT studies [7,8] responders from this analysis excluded those who achieved remission. The above provides the separation of these two states in a model.

In PURSUIT-SC study, the effectiveness was assessed at week 6 (after induction treatment) [7], while in PURSUIT-M study the assessment was made at week 30 and at week 54 (maintenance treatment; data available only for week 54 [8]). Transition probabilities for golimumab/standard care after discontinuation of the biological treatment were assumed to be the same as for the standard care alone arm. Clinical parameters and utility values used in the model are presented in Table 1.

To derive the probability of colectomy, we used data from study by Feagan et al. [9] for adalimumab, as no data concerned the colectomy rate after golimumab treatment was identified. It was estimated that during the 52-week period 3.68% (15 per 408.1 patient-years) and 4.75% (11 per 231.7 patient-years) of patients treated with adalimumab/standard care and standard care alone have a colectomy, respectively. Using the above data as our basis we calculated the probability of a colectomy in one cycle (Table 1), assuming that results for golimumab/standard care are the same as for adalimumab/standard care.

The probability of surgery complications was calculated based on the study by Arai et al. [10] and Fazio et al. [11] (Table 1). After resolving the complications within the 8-week period, it was assumed that patients achieve post-surgical remission, just as patients who didn't experience any complications.

### Costs

The costs were presented in 2016 Polish zloty (PLN) and the results were presented in both PLN and Euros (€; €1 = 4.2 PLN). Direct medical costs considered in the model included those related to initiation and maintenance treatment with golimumab (drug and administration costs), standard care, monitoring and hospitalization cost, surgery costs, and treatment of complications after surgery costs.

The costs of drugs used in the study population are based on the actual unit prices of reimbursement medical products. Table 2 presents all drugs' unit costs. The dosage of drugs used in standard care, as well as monitoring costs were determined by expert opinion.

Indirect costs come from study carried out in Poland on 202 patients with UC (S1 File). They include absenteeism, presenteeism and costs of leaving earlier the labor market, separately for remitted patients and those with active disease. Indirect costs generated by patients in remission were assigned to responders (patients with respond or remission) and indirect costs generated by patients with active disease were assigned to the rest.

### Utilities and quality of life

A systematic review was made to identify the utility values for different health states in the model. After analysis of the available data we chose the values presented by Woehl et al. [12] because this study is the most useful for source utility values of different stages included in the model, it reported EQ-5D utility values and was carried out on 18,573 patients from United Kingdom (Table 1). The utility values were reported for following states: remitting disease, mild disease, and moderate to severe disease. These categories of disease severity were based on the Simple Colitis Activity Index. We assumed that the utility value for moderate to severe disease that responded to treatment was equal to the value for mildly active disease by Woehl et al. [12]. In patients during the treatment or who are in an active disease state or had complications after surgery, the utility value was assumed to be as in active moderate to severe disease. We assumed that in the post-surgery remission state the utility value would be lower than in remission after the treatment state, which reflects the effect of chronic complications after a colectomy on the patient's quality of life. All utility values are presented in Table 1.

An alternative set of utility values was used in the sensitivity analyses, based on the study by Arseneau et al. [13]. There was no change in the sensitivity analyses in utility value for remission after surgery state, to all other states different utility values were assigned and are presented in Table 1.

### Economic analysis

The primary outcome of this simulation study was the ICUR of the treatment with golimumab/standard care and the standard care alone, expressed as an incremental cost per QALY saved. The ICUR was calculated as the difference in total costs from the public payer's perspective (only direct costs included) and social perspective (direct and indirect costs included) divided by the difference in effectiveness in QALYs.

### Sensitivity analysis

Sensitivity analyses to evaluate the robustness of our findings were conducted. Variability of cost-effectiveness results according to the change of key variables was assessed using one-way sensitivity analysis. Values used in sensitivity analysis for clinical, cost and utility parameters are presented in Tables 1. and 2. Additionally, within the sensitivity analysis, we assumed no limitation of golimumab treatment (biologic therapy until disease progression, i.e. lose of response, or death) and higher maintenance dose of golimumab—100 mg.

Parameter uncertainty was evaluated using probabilistic sensitivity analysis (PSA). We used normal (for cost and clinical parameters), log-normal (for clinical parameters), and beta (for utility weights) distributions.

## Results

### Base-case analysis

The results of the base-case analysis are presented in Table 3. The treatment of UC patients with golimumab/standard care instead of the standard care alone resulted in 0.122 additional years of life in full health.

**Table 3:** Base-case results.

End point	Golimumab + standard care	Standard care alone	Incremental value
QALY	19.241	19.118	0.122
Total direct costs—public payer perspective^	93,321 PLN	45,502 PLN	47,819 PLN
Total direct and indirect costs—social perspective^^	302,848 PLN	257,092 PLN	45,757 PLN
ICUR—public payer perspective	391,252 PLN/QALYG		
ICUR—social perspective	374,377 PLN/QALYG		

^ total direct costs include: pharmacotherapy costs (biological treatment), standard care costs, monitoring costs, golimumab administration costs, colectomy and complications after surgery costs

^^ total indirect costs included absenteeism, presenteeism, cost of early leaving the labor market.

PLN—Polish zloty, €1 = 4.2 PLN, based on the average exchange course from the year 2015

The treatment with golimumab/standard care was found to be more expensive than treatment with the standard care alone from the NHF perspective by 47,819 PLN (€11,386) and from the social perspective by 45,757 PLN (€10,894). The incremental cost per QALY gained was 391,252 PLN (€93,155) from NHF perspective and 374,377 PLN (€89,137) from social perspective (Table 3.).

### Sensitivity analysis

Results of various one-way sensitivity analyses are presented in Table 4. The range of ICUR values obtained during the one-way sensitivity analysis was from 211,990 PLN/QALYG (50,474 €/QALYG) to 959,633 PLN/QALYG (228,484 €/QALYG) from NHF perspective and from 194,071 PLN/QALYG (46,207 €/QALYG) to 946,069 PLN/QALYG (225,255 €/QALYG) from social perspective. The high influence on the analysis results has a change of remission rates after induction treatment (week 6) and relative risk of surgery rate (upper value). We also observed that change of utility value for remission state significantly affected the results. Biological treatment with no time restriction (until disease progression, i.e. lose of response, or death) resulted in ICUR value equals 701,517 PLN/QALYG (167,028 €/QALYG) from NHF perspective and 697,798 PLN/QALYG (166,142 €/QALYG) from social perspective. The difference in QALY between golimumab/standard care and standard care alone was 0,255 with assumption of no limitation of biological treatment. Assuming the maintenance dose of golimumab equals 100 mg, the ICUR value was 586,586 PLN/QALYG (139,663 €/QALYG) from NHF perspective and 569,274 PLN/QALYG (135,541 €/QALYG) from social perspective.

**Table 4:** Sensitivity analysis results.

Parameter	ICUR [PLN/QALYG]	
	Public payer perspective	Social perspective
Time horizon—lower value (10 years)	327,428	298,629
Time horizon—upper value (50 years)	394,061	377,954
Discount rate for outcomes—lower value (0%)	395,960	378,882
Discount rate for outcomes—upper value (5%)	389,761	372,950
Discount rate for costs—lower value (0%)	392,850	379,656
Discount rate for costs—upper value (10%)	389,883	370,234
Response—6. week, golimumab/standard care (RR)—lower value	354,712	339,232
Response—6. week, golimumab/standard care (RR)—upper value	444,816	425,897
Response—6. week, standard care alone—lower value	367,131	350,839
Response—6. week, standard care alone—upper value	419,370	401,817
Remission—6. week, golimumab/standard care (RR)—lower value	959,633	946,069
Remission—6. week, golimumab/standard care (RR)—upper value	211,990	194,071
Remission—6. week, standard care alone—lower value	583,193	567,711
Remission—6. week, standard care alone—upper value	280,549	262,871
Response—54. week, golimumab/standard care (RR)—lower value	391,252	374,377
Response—54. week, golimumab/standard care (RR)—upper value	391,252	374,377
Response—54. week, standard care alone—lower value	392,686	375,807
Response—54. week, standard care alone—upper value	389,159	372,291
Remission—54. week, golimumab/standard care (RR)—lower value	391,252	374,377
Remission—54. week, golimumab/standard care (RR)—upper value	391,252	374,377
Remission—54. week, standard care alone—lower value	305,976	288,676
Remission—54. week, standard care alone—upper value	492,087	475,472
Complications after surgery—lower value	391,523	374,621
Complications after surgery—upper value	391,252	374,377
Utility weight, active treatment—lower value	356,528	341,151
Utility weight, active treatment—upper value	391,252	374,377
Utility weight, remission—lower value	509,035	487,080
Utility weight, remission—upper value	363,237	347,570
Utility weight, remission after surgery—lower value	391,252	374,377
Utility weight, remission after surgery—upper value	391,252	374,377
Utility weight, complications after surgery—lower value	391,252	374,377
Utility weight, complications after surgery—upper value	391,403	374,522
Utility weight, response—lower value	452,879	433,346
Utility weight, response—upper value	344,389	329,535
Maximal treatment duration—lower value (12 months)	391,252	374,377
Maximal treatment duration—upper value (360 months)	701,517	697,798
Surgery rate (RR)—lower value	334,186	321,837
Surgery rate (RR)—upper value	676,080	636,619

PLN—Polish zloty, €1 = 4.2 PLN, based on the average exchange course from the year 2015; RR—relative risk; QALYG—Quality adjusted life years gained; ICUR—incremental cost-utility ratio.

**Fig 2:**
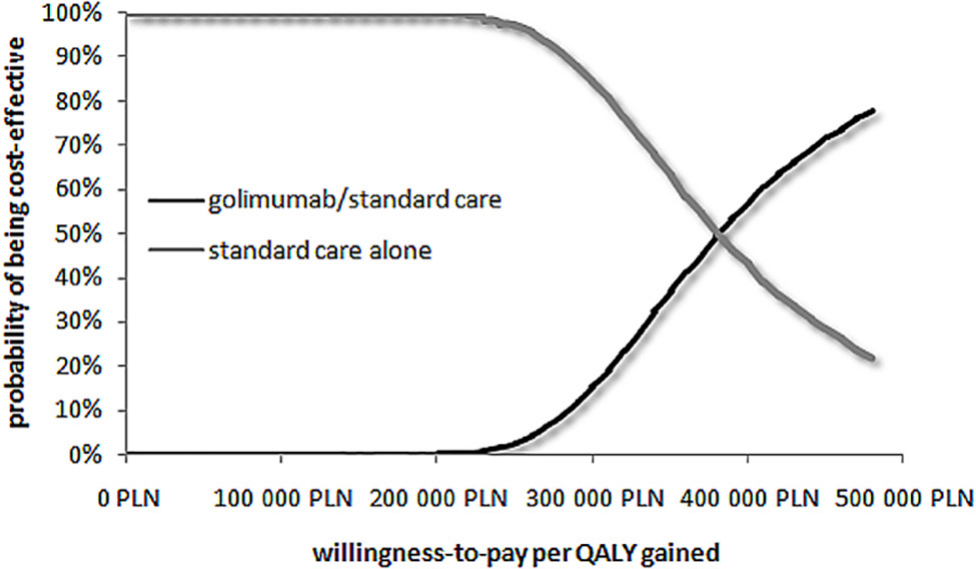
Cost-effectiveness acceptability curve showing the probability that golimumab with standard care is cost-effective versus standard care alone at a range of different threshold values.

The results of the Probabilistic Sensitivity Analyses, testing the whole range of all the uncertain parameters, are presented as a cost-effectiveness acceptability curve (Fig 2). The mean ICUR and the 95% confidence interval for golimumab/standard care when compared to the standard care alone was 379,685 PLN/QALYG, 95% CI: 246,281–915,800 (90,401 €/QALYG, 95% CI: 58,638–218,048) from the public payer’s perspective and 362,523 PLN/QALYG, 95% CI: 230,463–881,184 (86,315 €/QALYG, 95% CI: 54,872–209,806) from social perspective. The results of the PSA suggest golimumab/standard care to be cost-effective with a WTP equals about 380,000 PLN/QALYG (~ €90,476).

## Discussion

Using a 30 years’ time horizon and the restriction for the duration of TNFα inhibitor therapy to 1 year, golimumab/standard care treatment turned out to be more effective and more costly option compared with the standard care alone in Poland. One year biologic treatment provided an ICUR value of 89,137–93,155 €/QALYG, depending on the perspective.

The present economic model is the first study which assesses the golimumab treatment of UC in Poland with indirect costs of the disease included. We used data from randomized clinical trials to assess the effectiveness of biological treatment, as there is no data concerned Polish patients. Such a model can offer support to the decision makers as long as it reflects real-world conditions. Even though the present study evaluated the cost-effectiveness of golimumab in the Polish setting, the results could be easily adopted by the healthcare system in other countries. As the clinical practice (the length of therapy, dosage, indications and contradictions) is commonly based on clinical studies, the only difference should be the cost of drugs, medical procedures and hospitalization.

The limitation of our study is that we didn't include mortality due to UC because there is an evidence which indicates that patients with UC have normal life expectancy, subsequently meaning that treatment will not influence the survival. Additionally, mortality was not an outcome in the reference clinical studies. We also didn't include treatment related adverse events, as they have a relatively small impact on the cost and quality of life. Some assumptions had to be made concerning the utility values. The utility value for mildly active disease was assigned to patients who responded to treatment, while in patients who had complications after surgery, the utility value was assumed to be as that in active moderate to severe disease.

Review of published economic analysis for study subject was performed. We found two studies for golimumab/standard care compared with standard care alone in UC. Canadian Agency for Drugs and Technologies in Health (CADTH) published a recommendation for golimumab in UC [14]. Within this recommendation the results of economic analysis carried out by the manufacturer were presented. It was reported that when compared with conventional therapy in 10-years time horizon, golimumab 50 mg and 100 mg were associated with an ICUR of $41,591 (€37,349) and $42,271 (€37,959), respectively. Agency pointed out the key limitation of the manufacturer's economic evaluation which was the lack of transparency regarding its methods and how data were included in the model. Agency made their own assessment for 1.25 and 2.5 years time horizon and obtained ICUR values of $104,000 (€93,392) and $52,000 (€46,696), respectively. Another identified economic evaluation [15] was presented in a form of abstract, full text is not available. Thorlund et al [15] conducted the cost-effectiveness analysis of three anti-TNF-alfa inhibitors (infliximab, adalimumab and golimumab). Markov model was used and the analysis was performed in 10 years time horizon from the Canadian public payer's perspective, considering direct costs only. The ICUR for golimumab was of approximately $40,000 (about €35,920). Golimumab provides a cost-effective treatment option for patients who have had an inadequate response to conventional therapy for moderately to severely active ulcerative colitis. As in the case of previous study, it is hard to compare our results with above, because only abstract or technology assessment report with limited information about methodology, assumptions, and inputs is available. Published economic analysis showed that golimumab/standard care compared with standard care alone in UC seems to be an cost-effective treatment option, with ICUR value ranged from €35,920 to €93,392. It is worth to mention that none of the identified studies included indirect costs.

During the review of published economic analysis for biologic therapies used in UC, we also found the assessment for infliximab, made by Xie et al [16]. Analysis was carried out for Canada in 5 years’ time horizon. Authors assumed switching to adalimumab therapy after the failure of infliximab. The ICUR for the biologic treatment strategy (infliximab and adalimumab after failure of the first biologic therapy) compared with usual care was about 306,761 USD (2015 prices). The QALYs for the biologic treatment strategy were 2.178, and for the usual care strategy—2.015 (QALYG = 0.163). The difference in QALYs between the compared treatment options was above the value obtained in our analysis for golimumab/standard care compared with standard care alone (0.122), which may indicate the higher additional effect of infliximab compared with standard care than of golimumab/standard care compared with standard care alone. Above conclusion should be interpreted with caution as the control groups in studies for infliximab and golimumab, as well as utility values, time horizon and model structure can differ significantly.

Some economic evaluations for adalimumab were also found, but all of them were available only in a form of abstract where limited information was provided. In this connection it was not possible to compare the clinical effects with those obtained in our analysis.

Our analysis has shown that despite the clinical advantage of biologics, its use constitutes a heavy burden for the public payer. The treatment of UC patients with golimumab/standard care in place of a standard care alone resulted in additional quality adjusted life years but also additional costs. The biologic therapy remains the only treatment option for moderate to severe chronic UC patients who failed standard treatment or when it is not tolerated.

## Supporting Information

S1 File Disease activity, quality of life and indirect costs of ulcerative colitis in Poland. (DOC)
