# Supplementary material for: Cost-Effectiveness Analysis of 1-Year Treatment with Golimumab/Standard Care and Standard Care Alone for Ulcerative Colitis in Poland
Source: PLoS One. 2016 Aug 5;11(8):e0160444. doi: 10.1371/journal.pone.0160444 (PMC4975491; doi:10.1371/journal.pone.0160444)
Supplement: S1 File — (DOC) [file pone.0160444.s001.doc]

**DISEASE ACTIVITY, QUALITY OF LIFE AND INDIRECT COSTS OF ULCERATIVE COLITIS IN POLAND**

A questionnaire-based survey was used to collect data on disease activity, health-related quality of life and productivity loss of UC patients in Poland. The survey was started in October 2015 and has been planned to be finalized at the end of the first quartile of 2016. The results presented in this study are preliminary with a cut-off date of 31 December 2015, when a threshold population of the above 200 patients was reached. The study was performed in a group of patients with a diagnosis of UC, in cooperation with the Polish Association Supporting People with Inflammatory Bowel Disease “J-elita” (j-elita.org.pl). Patients filled in the paper version of the questionnaire regarding the disease activity index (P-SCCAI), comorbidities, prescribed treatment for UC, basic characteristics (age, sex, work status, date of disease onset, place of living, working status), disability status (EQ-5D-3L), expenses on medical and non-medical resources (e.g., private medical consultation, over-the-counter medication and dietary supplements, information materials) and loss of productivity (presenteeism, absenteeism), informal care, and benefits from a social care institution due to UC. Respondents were allowed to return the questionnaire by traditional mail, e-mail (scanned version) or saved directly on the server dedicated for this study (scanned version).

To assess the health-related quality of life, the EuroQol 5 dimensions 3 level version (EQ-5D-3L) questionnaire was used^1^ and it was evaluated with the Polish norms presented by Golicki et al.^2^ The EQ-5D-3L takes into account five most important aspects of life: mobility, self-care, housekeeping activities, pain, and anxiety. For each domain patient can report the lack of problems (represented by digit 1), the occurrence of some problems (digit 2) and the occurrence of many problems (digit 3). Data obtained using the EQ-5D-3L questionnaire can be converted into utility using population tariffs.^3^

The WPAI questionnaire^1^ was the starting point for the preparation of a part of the questionnaire concerning loss of productivity (presenteeism, absenteeism), usual activity impairment, informal care, and benefits from a social care institution; it was modified to adapt the questionnaire to part-time and seasonal employees, patients on rehabilitation benefit or other temporally inactive patients or those awaiting the assessment of incapacity for work.

The HCA was used to estimate indirect costs due to absenteeism and presenteeism. Two macroeconomic indicators for Poland were considered: gross domestic product per capita (GDP, €10,688 or 44,677 PLN) and gross value added (GVA, €25,055 or 104,728 PLN) per worker presented in 2014 prices in euro (the exchange rate used was 1 euro = 4.18 PLN, which was an average exchange rate in 2015) with a correction factor of 0.65 (the conventional mean value of output elasticity of labour according to the Cobb-Douglas function of production; this approach is suggested by the European Commission because increasing the amount of a single factor of production, with all other factors of production constant, decreased the marginal output of a production).^4, 5^ The GDP per capita is a commonly used measure of a country’s economic development. Its weakness is that it considers not only employees but also people who are economically inactive, such as children and retirees. It was calculated by dividing the GDP for Poland in the year 2014 (1,719,097 million PLN) by the Polish population in the same year (38,478,600; above values come from the Central Statistical Office of Poland, www.stat.gov.pl). The GVA per worker reflects true economic development in areas of production. It is expressed as value per worker and does not consider economically inactive people. The GVA per worker has been recognized as the most appropriate measurement for examining the decrease in human capital due to illness, as it represents the productivity of a country in all economic sectors. It was calculated by dividing the GVA for Poland in 2014 (1,525,193 million PLN) by the Polish population in the same year.

Continuous variables were summarised using means and standard deviations (SDs) or median and interquartile range (IQR), while nominal variables were summarised using counts and percentages. The Spearman’s correlation was used to present the association between disease activity and absenteeism as well as presenteeism. P-values of less than 0.05 indicated statistical significance. Additionally, data on disease activity and generated indirect costs were presented on scatterplots. Absenteeism was presented as a number of days missed from work per year, while presenteeism was expressed as a percentage of standard work efficiency achieved due to UC. Statistical analyses were performed using STATISTICA®.

**Results**

We obtained 202 completed questionnaires from patients aged from 18 to 71 years (average age was 33.14 years, SD: 9.90; median value was 31.00 years). The basic characteristics of the population are presented in Table 1.

**Table 1**. Basic characteristics of respondents included in the study

| Characteristic | | N | Value |
| --- | --- | --- | --- |
| Age [years] | | 202 | 33.14 (SD: 9.90), range:18-71 |
| Male | | 198 | 75 (37.88%) |
| Disease onset [years] | | 201 | 26.35 (SD: 8.89), range:8-62 |
| Place of living | City < 100,000 citizens | 200 | 60 (30.00%) |
|  | City ≥ 100,000 citizens |  | 119 (59.50%) |
|  | village |  | 21 (10.50%) |

Of 202 patients, 190 (94.1%) provided data on disease activity with the P-SCCAI questionnaire. Based on those values, the mean P-SCCAI score was 8.26 (SD: 5.26; range: 0 - 24). Among 190 patients with UC, 26.3% had remission according to the P-SCCAI score. Patients also assessed disease activity on their own, based on the clinician's opinion during the last consultation. The compliance of patient's own assessment with the P-SCCAI score was 53.4%.

Patients reported between 0 and 10 consultations with a clinician during the month before completing the questionnaire with a mean value of 1.22 (SD: 1.71), of which 62.12% were private visits with an average cost of the consultation of 141.69 PLN (SD: 59.99).

About 55.5% of patients spent from 0 to 100 PLN monthly on drugs prescribed by the clinician, and only 3.47% spent more than 600 PLN monthly. The similar amount of money was spent on dietary supplements and other medicines that are not prescribed by the clinician (over-the-counter drugs). The smallest amount of money was spent monthly on information materials about the disease: more than 90% of patients spent from 0 to 100 PLN and no patient spent more than 400 PLN.

Almost 96% of patients (193 of 202) provided information about the drugs used. The vast majority of patients (92.23%) took mesalazine. The other often administered drugs were: azathioprine (26.94%), prednisone or prednisolone (21.24%), and sulfasalazine (13.47%). Only 4.66% of patients declared to use biologic drugs: adalimumab (2.59%) and infliximab (2.07%). Only 11 patients had surgery due to UC, which was restorative proctocolectomy in the majority of cases.

A number of UC patients (27.8%) required help with daily activities; in 95.5% of cases, the help was provided by family members. Only 11.2% of patients received financial benefits from state institutions in a form of disability pension, social pension, illness or rehabilitation benefit, and 8.4% received a financial support from other sources.

Data on employment status was collected for 202 patients, of which 150 (74.26%) were currently working. Among all working patients, 130 were on full-time contract. Thirty-four patients (16.83%) were still studying and 6.44% did not have paid employment. Only 7 patients were on pension due to UC and 7 were unable to work. The average monthly amount of social pension was 605.75 PLN, and it was slightly lower than pension due to inability to work (681.25 PLN).

The average annual number of days off work due to illness was 30.49 (SD: 65.37; range: 0 - 279). Most people (65.31%) reported lack of days off work due to the disease.

The relationship between absenteeism and disease activity was moderate (correlation of 0.4603 with a P-value of less than 0.05).

Average on-the-job productivity loss was 19.38% (SD: 23.64%; range: 0 - 100%), which represents the extent of presenteeism. Only 4.1% of working patients reported on-the-job productivity loss of at least 70%, while 44.8% of working patients reported no on-the-job productivity loss (value 0). Data were presented for 145 working patients with UC; five working patients did not provide data on on-the-job productivity loss.

The relationship between presenteeism and disease activity was moderate (correlation of 0.4194 with a P-value of less than 0.05, which indicates statistical significance). Loss of on-the-job productivity was positively correlated with disease activity.

Using the GDP per capita, the indirect costs of absenteeism per working person were €679 (SD: €1,691) or 2,837 PLN (SD: 7,070 PLN), and the indirect costs of presenteeism per working person were 1,346 (SD: €1,642; median €695, IQR: €1,642) or 5,628 PLN (SD: 6,864 PLN; median 2,904 PLN, IQR: 6,864 PLN). Taking into account the GVA per worker, the costs were as follows: €1,591 (SD: €3,965) or 6,651 PLN (SD: 16,573 PLN) for absenteeism, and €3,156 (SD: €3,849; median €1,629, IQR: €3,849) or 13,192 PLN (SD: 16,089 PLN; median 6,807 PLN, IQR: 16,089 PLN) for presenteeism.

The average annual indirect cost of both absenteeism and presenteeism per working person was €2,043 (SD: 2,876; median €1,389, IQR: 3,215) or 8,543 PLN (SD: 12,020; median 5,808 PLN, IQR: 13,441) calculated using the GDP and €4,791 (SD: 6,741; median €3,257, IQR: 7,537) or 20,026 PLN (SD: 28,178; median 13,615 PLN, IQR: 31,507) calculated using the GVA. Data were based on the answers of 144 working patients with UC. The cost of presenteeism accounted for 65.87% of the total indirect costs.

As a sensitivity analysis, the results without the correction factor (marginal productivity of labour) were presented. Total annual indirect costs per patient were €2,989 (SD: €3,801) or 12,495 PLN (SD: 15,887 PLN), as calculated using the GDP, and €7,007 (SD: €8,910) or 29,289 PLN (SD: 37,242 PLN), as calculated using the GVA.

The relationship between indirect costs calculated using the GDP or GVA and disease activity was moderate (correlation of 0.4651 with P-value of less than 0.05).

Data on the quality of life were also reported by almost all respondents (201 of 202; 99.5%). Based on data from Table 2, the health states were created. The most common state was “11122”, reported by 58 patients (28.86%). The highest (worse) state observed was “22222” and occurred in 3 patients (1.49%). Based on these data the utility was calculated using the Polish tariff.^14^ The mean utility equalled 0.8651 (SD: 0.1285; range: 0.1720 - 1).

**Table 2**. Number of patients reporting lack of problems, some problems or a lot of problems in specific life domains taken into consideration in the EQ-5D-3L questionnaire.

| Life domain | Problems reported with specific life domains | | |
| --- | --- | --- | --- |
|  | No | Some | A lot |
| Mobility | 179 (89.05%) | 22 (10.95%) | 0 (0%) |
| Self-care | 197 (98.01%) | 4 (1.99%) | 0 (0%) |
| Usual activities | 149 (74.13%) | 49 (24.38%) | 3 (1.49%) |
| Pain | 69 (34.33%) | 127 (63.18%) | 5 (2.49%) |
| Anxiety | 67 (33.33%) | 124 (61.69%) | 10 (4.98%) |

**References**

1. EuroQol. http://www.euroqol.org/ Last Access: October 2015.
2. Golicki D, Jakubczyk M, Niewada M, Wrona W, Busschbach JJ. Valuation of EQ-5D health states in Poland: first TTObased social value set in Central and Eastern Europe. Value Health 2010; 13: 289-97.
3. Krol M, Brouwer W. How to Estimate Productivity Costs in Economic Evaluations. PharmacoEconomics 2014; 32: 335–44.
4. Central Statistical Office of Poland, <http://stat.gov.pl/en/> Last Access: January 2016.
5. Havik K, Mc Morrow K, Orlandi F, et al. The production function methodology for calculating potential growth rates and output gaps. Economic Papers 535, November 2014, http://ec.europa.eu/economy_finance/publications/economic_paper/2014/pdf/ecp535_en.pdf.
